# Monitoring mosaic biotopes in a marine conservation zone by autonomous underwater vehicle

**DOI:** 10.1111/cobi.13312

**Published:** 2019-04-29

**Authors:** Noëlie M.A. Benoist, Kirsty J. Morris, Brian J. Bett, Jennifer M. Durden, Veerle A.I. Huvenne, Tim P. Le Bas, Russell B. Wynn, Suzanne J. Ware, Henry A. Ruhl

**Affiliations:** ^1^ Ocean Biogeochemistry and Ecosystems National Oceanography Centre Southampton SO14 3ZH U.K.; ^2^ University of Southampton Southampton SO14 3ZH U.K.; ^3^ University of Hawaii Honolulu HI 96822 U.S.A.; ^4^ Centre for Environment Fisheries and Aquaculture Science Lowestoft NR33 0HT U.K.

**Keywords:** benthos, biotope classification, ecological metrics, marine protected area, mosaic habitats, seafloor, área marina protegida, bentos, clasificación de biotopos, fondo marino, medidas ecológicas, mosaico de hábitats, 海洋保护区, 海底, 海底生物, 群落生境分类, 镶嵌型生境, 生态指标

## Abstract

The number of marine protected areas (MPAs) has increased dramatically in the last decade and poses a major logistic challenge for conservation practitioners in terms of spatial extent and the multiplicity of habitats and biotopes that now require assessment. Photographic assessment by autonomous underwater vehicle (AUV) enables the consistent description of multiple habitats, in our case including mosaics of rock and sediment. As a case study, we used this method to survey the Greater Haig Fras marine conservation zone (Celtic Sea, northeast Atlantic). We distinguished 7 biotopes, detected statistically significant variations in standing stocks, species density, species diversity, and faunal composition, and identified significant indicator species for each habitat. Our results demonstrate that AUV‐based photography can produce robust data for ecological research and practical marine conservation. Standardizing to a minimum number of individuals per sampling unit, rather than to a fixed seafloor area, may be a valuable means of defining an ecologically appropriate sampling unit. Although composite sampling represents a change in standard practice, other users should consider the potential benefits of this approach in conservation studies. It is broadly applicable in the marine environment and has been successfully implemented in deep‐sea conservation and environmental impact studies. Without a cost‐effective method, applicable across habitats, it will be difficult to further a coherent classification of biotopes or to routinely assess their conservation status in the rapidly expanding global extent of MPAs.

## Abstract 2

*Article impact statement*: AUVs offer a solution to monitoring challenges posed by the mixed habitats and growing spatial extent of marine protected areas globally.

## Abstract (es)

Monitoreo de Mosaicos de Biotopos en una Zona Marina de Conservación por medio de un Vehículo Submarino Autónomo

**Resumen**

El número de áreas marinas protegidas (AMP) ha incrementado dramáticamente en la última década y ahora presenta un gran reto logístico para quienes practican la conservación en términos de extensión espacial y la multitud de hábitats y biotopos que ahora requieren ser evaluados. La evaluación fotográfica por medio de vehículos submarinos autónomos (VSA) habilita la descripción constante de múltiples hábitats, y en nuestro caso incluso mosaicos de rocas y sedimento. Como estudio de caso usamos este método para censar la zona marina de conservación de Greater Haig Fras (Mar Celta, noreste del Atlántico). En él distinguimos siete biotopos; detectamos variaciones estadísticamente significativas en el stock permanente, la densidad de especies, la diversidad de especies y la composición faunística; e identificamos especies indicadoras significativas para cada hábitat. Nuestros resultados demuestran que la fotografía basada en VSA puede producir datos sólidos para la investigación ecológica y la conservación marina práctica. La estandarización a un número mínimo de individuos por unidad de muestreo, en lugar de a un área fija del fondo marino, puede ser un recurso valioso para definir una unidad de muestreo ecológicamente adecuada. Aunque el muestreo compuesto representa un cambio en la práctica habitual, otros usuarios deberían considerar los beneficios potenciales de esta estrategia en los estudios de conservación. Este método puede aplicarse de manera generalizada en el ambiente marino y ha sido implementado exitosamente en la conservación y en estudios de impacto ambiental en mares profundos. Si no existe un método rentable, aplicable en todos los hábitats, será difícil avanzar hacia una clasificación coherente de los biotopos o hacia una evaluación rutinaria de su estado de conservación en la extensión mundial de rápida expansión de las AMP.

## 摘要

海洋保护区的数量在过去十年中急剧增加, 这在空间尺度和目前需要评估的物种生境及群落生境多样性的方面, 对保护工作者的管理工作都提出了重大挑战。自动潜航器的影像评估可以对多种生境类型进行统一的描述, 在本研究的案例中就包括含有岩石和沉积物的镶嵌型生境。本研究用上述方法调查了位于大西洋东北部凯尔特海的 Greater Haig Fras 海洋保护区。我们区分出七种群落生境, 它们在物种总量、物种密度、物种多样性和动物群组成上都有统计学上显著的差异; 我们同时也找出了每种生境的重要指示性物种。研究结果表明, 自动潜航器拍摄的影像可以为生态学研究和海洋保护实践提供可靠的数据。对每个采样单位的最小个体数进行标准化 (而不是对固定的海底区域进行标准化), 可能是确定合适的生态学采样单位的重要方法。虽然这种综合采样的方法在标准做法的基础上有所改变, 但其他使用者也应考虑到它在保护研究中潜在的好处。这个方法广泛适用于各种海洋环境, 并已成功应用于深海保护和环境影响研究。如果没有这样一种适用于不同生境的经济有效的方法, 就很难进一步对群落生境进行统一分类, 也难以定期评估全球迅速扩增的海洋保护区的保护现状。**【翻译: 胡怡思; 审校: 聂永刚】**

## Introduction

Acquiring ecological data is key to basic biological research, monitoring change in biodiversity, and the development of effective conservation actions. Achieving those aims in a timely and cost‐effective manner remains a significant challenge in terrestrial and aquatic systems. In both cases, drones—unmanned aerial vehicles (UAVs) and autonomous underwater vehicles (AUVs)—promise significant advances in capability (Anderson & Gaston 2013; Wynn et al. 2014).

Marine protected areas (MPAs) have long been suggested as a tool for maintaining and restoring biodiversity (Woodcock et al. 2017), and the designation of numerous MPAs is now driving the need for better and more cost‐effective description and quantification of the biological assemblages present and their habitats. Autonomous underwater vehicles are an established technology in seafloor research (Durden et al. 2016c) and appear to be an effective tool in science‐ and conservation‐driven studies both in shelf‐sea (Marzinelli et al. 2015) and deep‐sea (Morris et al. 2016) environments . They offer rapid, nondestructive data collection, access to a wide range of habitats, and reduced survey costs (Wynn et al. 2014). Data from AUVs can improve the quantification of conservation metrics (Durden et al. 2016a) and may be of particular value in habitats where remote sampling methods are ineffective, such as reef or rock habitats (Tolimieri et al. 2008).

Typically, MPAs encompass multiple habitats, and the use of various samplers (e.g., grabs, trawls, towed cameras) has limited the degree to which the resultant data can be synthesized across substratum types. The European Nature Information System (EUNIS) provides a classification of habitats and biotopes that has been influential in standardizing habitat description (Costello 2009), although its limitations have become evident as conservation‐based marine mapping has expanded. In particular, important mixed, or mosaic, marine habitats “cannot be represented using the current EUNIS classification system as it only recognizes separate rock or sediment habitats” (Galparsoro et al. 2012: 2634). Mosaic habitats likely play a key role in the connectivity that underpins the functioning of MPA networks (Olds et al. 2016), and how they might best be classified remains an area of active debate (Dauvin 2015). It is the rule‐based hierarchical nature of EUNIS (e.g., rock or sediment) that poses the problem, which may similarly impact other hierarchical systems (Harris 2012).

Where habitat‐type‐dependent field methods are employed, a single biotope classification scheme can be difficult or impossible to operate (Van Rein et al. 2009). Different field methods also introduce major mismatches in both the spatial scale observed and the corresponding body sizes and taxonomic groups assessed. These difficulties could be reduced and the full potential of AUV‐based monitoring realized if visual assessment by photography could be implemented usefully across multiple biotopes. The benefits include use of common scales and methods across habitats, and consequently a common classification scheme; explicit recording of the species and habitats that underpin MPA designation and legislation; and direct evidence of violating activities from indicators such as trawl marks and human debris. However, as Galparsoro et al. (2012) indicate, 2 questions remain: how robust are visually based classifications and what constitutes an appropriate sampling unit in photographic assessments?

**Figure 1:**
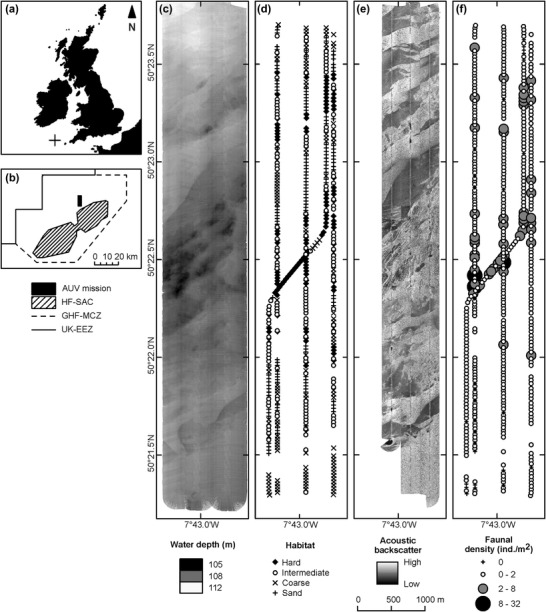
(a) Location of the Greater Haig Fras marine conservation zone (GHF‐MCZ) in the Celtic Sea, (b) area of autonomous underwater vehicle survey and adjacent Haig Fras special area of conservation within the GHF‐MCZ, (c) bathymetry, (d) photographic habitat classification (hard, ≥50% seafloor cover by bedrock, boulder, cobbles; intermediate, ≥10% seafloor cover by bedrock, boulder, cobbles; coarse, >90% seafloor cover by gravelly sand, granules, pebbles, shells; sand, >90% seafloor cover by sand), (e) sidescan sonar backscatter intensity, and (f) photographic estimate of faunal numerical density (combined invertebrates and demersal fish; >=1 cm body length).

To tackle these questions we undertook an AUV survey in the Greater Haig Fras marine conservation zone (MCZ) (Wynn et al. 2014) (Fig. 1a,b). Nested within the MCZ is the Haig Fras special area of conservation (SAC) that includes a bedrock outcrop reef. The MCZ has substantial areas of mixed rock‐sediment habitat that are difficult to assess by physical sampling. We used AUV data to investigate whether mosaic biotopes can be adequately described and discriminated on the basis of visual data; to establish potential links between biotope characteristics and substratum type and complexity, to demonstrate the potential effectiveness of the method; and to examine the influence of sampling unit choice in a practical conservation assessment of complex habitats.

## Methods

### Field Survey

All data were derived from a 16‐h deployment of the AUV *Autosub6000* in July 2012 (Ruhl 2013) during which the vehicle undertook 3 dives: swath bathymetry survey (dive 1) (Fig. 1c); photographic survey from a target altitude of 3.2 m above the seafloor with a Point Grey Research Grasshopper 2 camera (Morris et al. 2014) (dive 2); and a sidescan sonar survey (dive 3) (Fig. 1e). The swath bathymetry and sidescan sonar survey methods are detailed, but we used only data from the single photographic survey dive (duration 225 min) in our analyses here. The photographic survey was carried out as 4 north‐south transect lines and 1 crossing line (Fig. 1d,f) that targeted a rock outcrop of slightly elevated terrain (Fig. 1c) with sinuous striations in the sonar view (Fig. 1f).

### Image Data Generation

Images were processed to improve nonuniform illumination and color representation, rectified to a common scale (0.59 mm/pixel), georeferenced, and mosaicked into groups of 5 consecutive images (tiles) (Morris et al. 2014). In total, 2637 such tiles were produced, each representing approximately 7.3 m^2^ of seabed. Tiling was undertaken to remove overlap from consecutive photographs and as a practical convenience to reduce the data management overhead. Tiles were assessed in random order to avoid bias through knowledge of spatial proximity (Durden et al. 2016b). We present results from 3 distinct sampling units: tile (primary sampling element, physical scale approximately 7.3 m^2^, variable number of specimens); composite area (multiple tiles, approximately 150 m^2^, variable number of specimens); and composite individuals (multiple tiles, approximately 150 specimens, variable seabed area) (Table 1).

**Table 1:** Autonomous underwater vehicle photographic effort in the Greater Haig Fras marine conservation zone by habitat and substratum type

		Survey total				By composite‐area samples			By composite‐individuals samples		
Habitat type^a^	Substratum type^b^	no. of tiles^c^	area (m^2^)	area (%)	no. of individuals	no. of replicates	area (m^2^)^d^	no. of individuals^d^	no. of replicates	area (m^2^)^d^	no. of individuals^d^
Hard	H	121	882	4.6	2832	6	147	472	19	16	149
Hard	Hc	211	1564	8.1	3648	10	156	265	59	27	147
Hard	Hs	214	1656	8.6	4135	10	165	414	61	27	148
Intermediate	Ch	584	4255	22.1	1476	29	146	51	12	355	148
Intermediate	Sh	119	874	4.5	389	6	145	65	12	73	130
Coarse	C	669	4836	25.2	446	33	146	14	3	1612	149
Sand	S	719	5156	26.8	966	36	143	27	6	859	138
Mean							150	187		229	147
Total		2637	19223	100.0	12892	130			84		

a Definitions: hard, ≥50% seafloor cover by bedrock, boulder, cobbles; intermediate, ≥10% seafloor cover by bedrock, boulder, cobbles; coarse, >90% seafloor cover by gravelly sand, granules, pebbles, shells; sand, >90% seafloor cover by sand.

b Abbreviations: H, ≥50% seafloor cover by bedrock, boulder, cobbles; h, ≥10% seafloor cover by bedrock, boulder, cobbles; C, ≥50% seafloor cover by gravelly sand, granules, pebbles, shells; c, ≥10% seafloor cover by gravelly sand, granules, pebbles, shells; S, ≥50% seafloor cover by sand; s, ≥10% seafloor cover by sand.

c Tile, mosaicked set of 5 consecutive images.

d Mean of replicate values.

Three primary substratum types were recorded: hard substrata (bedrock, boulder, cobbles), coarse sediments (gravelly sand, granules, pebbles, shells), and sand. A primary substratum type was attributed based on majority tile area (>=50%), and a secondary type was recorded if present (≥10%). The combination of primary and secondary types yielded 4 mixed, or mosaic, substratum categories (e.g., Post et al. 2011) (Supporting Information). For presentation and analysis, the substratum classes were simplified into summary habitats (Table 1): hard habitats with hard primary substratum, intermediate habitats with hard secondary substratum, and coarse habitats and sand habitats (jointly referred to as sedimentary habitats) where hard substratum was absent. We did not observe a coarse or a sand mosaic habitat during the survey. Litter and other human debris on the seabed were also recorded (Supporting Information).

Invertebrates and demersal fish (>=1 cm body length) were counted, measured, and identified to the lowest taxonomic or morphotype unit possible (e.g., Althaus et al. 2015). For colonial and encrusting organisms, the greatest diameter of individual colonies, or patches, was measured. Solitary tubicolous polychaetes, bivalves, and gastropods were observed but excluded from the analyses to avoid inclusion of empty tubes or shells. Indeterminate specimens (<1% of total) were excluded from subsequent analyses. Body‐size measurements were converted to estimated gram wet weight (g wwt) biomass via existing length‐weight relationships (Supporting Information).

### Faunal Community Analysis

We considered the complete set of tiles represented the total statistical population (i.e., assessments were carried out within that population) and made no statistical inference beyond that population. Our primary objective was to test for biological differences between habitats; therefore, we first grouped the tiles by substratum type. In our case, and in many marine settings, a single photograph (or tile) was insufficient to establish a useful estimate of species diversity or composition. Consequently, we compiled data from multiple tiles to form our sampling units (replicates). Given the nonindependent nature of consecutive tiles and the inevitable occurrence of spatial autocorrelation (Legendre 1993), we compiled the data from individual tiles at random within substratum type to form composite‐area sampling units of approximately 150 m^2^/replicate (Table 1). A simplified illustration of this method and formal testing of the randomization process are in Supporting Information. We tested the effect of sampling unit choice in the same manner (composite‐individuals sampling units of approximately 150 individuals per replicate) (Table 1 & Supporting Information).

For density and biomass analyses, individual tile data were log‐transformed and assessed using Welch's 1‐way analysis of variance (ANOVA). Subsequent pairwise comparisons were made using the Games–Howell method, as implemented in Minitab (version 17) (Minitab, Coventry). To estimate density and biomass at physical scales greater than a single tile, data were repeatedly, randomly, accumulated with replacement to form larger physical samples of 2 to 724 tiles, and a median value was derived from the repeats, in R environment (R Core Team 2017).

For faunal diversity and composition analyses, replicate‐level data (composite area and composite individuals) (Table 1) were assessed. Faunal diversity was assessed by sample‐based rarefaction to estimate taxon richness (Sest) (Colwell et al. 2012), the exponential form of the Shannon index (expH´) (Magurran 2004), and the inverse form of Simpson's index (1/*D*) (Magurran 2004), as calculated via 1000 randomizations without replacement for Sest and with replacement for expH' and 1/*D* in EstimateS (version 9.1.0) (Colwell 2013). Faunal composition was assessed by 2‐dimensional nonmetric multidimensional scaling ordination based on the Bray‐Curtis dissimilarity of log‐transformed faunal density data and subsequent analysis of similarities (ANOSIM), all implemented using PRIMER (version 6.1.11) (Quest Research Limited, Auckland) (Clarke & Warwick 1994). Morphotype specificity and fidelity to particular substratum types was assessed by the indicator value method, as implemented in the R package indicspecies (De Cáceres & Legendre 2009), and by 2‐way indicator species analysis (TWINSPAN) (Hill 1979), as implemented in the software package PC‐ORD (version 4) (Wild Blueberry Media, Corvallis) with 5 logarithmically arranged density levels. To evaluate the choice of sampling unit, we produced autosimilarity curves (Schneck & Melo 2010), as employed by Durden et al. (2016b) in an assessment of seabed photography. The method calculates the average Bray–Curtis dissimilarity between pairs of composite samples formed from increasing numbers of tiles by random resampling of the original data within habitat type (1000 times without replacement; in R environment).

## Results

### Standing Stocks

**Figure 2:**
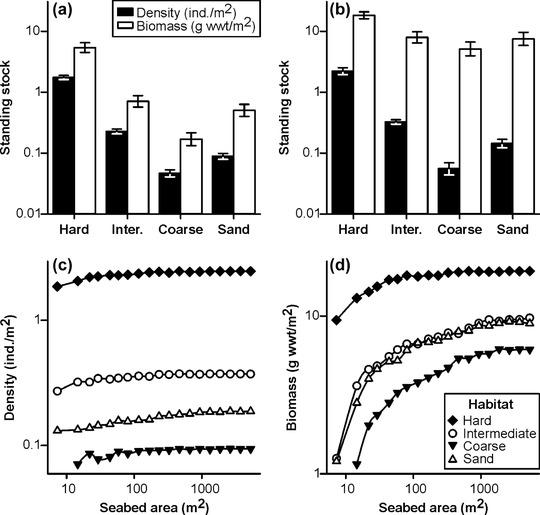
Standing stocks of combined invertebrates and demersal fish (>=1 cm body length) by summary habitat, as determined at (a) tile scale (approximately 7.3 m^2^) and (b) composite‐area sample scale (approximately 150 m^2^), illustrated as geometric mean values with corresponding 95% CIs. Additional illustrations of variation in estimated (c) median numerical density and (d) median biomass density, as determined from increasingly large seabed areas.

Whether assessed using tile‐level or composite‐area‐replicate data, faunal density exhibited a statistically significant difference between habitats (Welch's ANOVA, *p* < 0.001) (Fig. 2a,b); hard habitats had the highest density and coarse the lowest. All pairwise comparisons were significant (Games–Howell, *p* < 0.05). Area‐scaled density by habitat followed the same trends; apparent median density rapidly stabilized with seabed area assessed in all habitats (Fig. 2c). Faunal biomass also varied significantly between habitats when assessed using tile‐level data (Welch's ANOVA, *p* < 0.001) (Fig. 2a); hard habitats had the highest biomass and coarse the lowest. Pairwise comparisons indicated significant differences between all habitats (Games‐Howell, *p* < 0.05), except between intermediate and sand (Games–Howell, *p* = 0.14). When assessed using composite‐area‐replicate data, biomass also varied significantly between habitats (Welch's ANOVA, *p* < 0.001) (Fig. 2b); however, the magnitude of differences was substantially reduced. Pairwise comparisons indicated significant differences between all habitats (Games–Howell, *p* < 0.05), except between intermediate and sand (Games–Howell, *p* = 0.98) and between coarse and sand (Games–Howell, *p* = 0.15). Area‐scaled biomass by habitat followed the same trends; however, apparent median biomass was slow to stabilize with seabed area assessed. Estimated biomass in hard habitats stabilized at approximately 650 m^2^ and in other habitats at approximately 2000 m^2^ (Fig. 2d).

### Faunal Diversity

**Figure 3:**
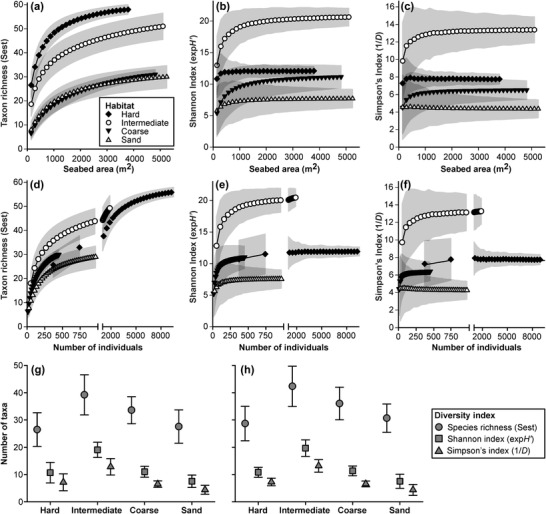
Sample‐based rarefaction of combined invertebrates and demersal fish (>=1 cm body length) morphotype diversity (taxon richness, exponential form of the Shannon index, and inverse form of Simpson's index) by habitat (hard, ≥50% seafloor cover by bedrock, boulder, cobbles; intermediate, ≥10% seafloor cover by bedrock, boulder, cobbles; coarse, >90% seafloor cover by gravelly sand, granules, pebbles, shells; sand, >90% seafloor cover by sand) as determined from (a–g) composite‐area samples and (h) composite‐individuals samples: (a–f) full rarefaction curves, (g) simplified results for composite‐area samples at an approximately equal number of individuals (364–375), and (h) simplified results for 3‐sample composite‐individuals samples case (number of individuals 446–483). In all plots, mean values and corresponding 95% CIs are shown (shaded areas and error bars, respectively).

Assessed by composite‐area replicates, taxon richness (Sest) exhibited statistically significant differences between habitats; hard and intermediate were notably richer than coarse or sand habitats (Fig. 3a). However, these differences were less clear‐cut when rarefied by number of individuals (Fig. 3d). In contrast, heterogeneity diversity (exp*H*') and dominance diversity (1/*D*) showed consistent, statistically significant differences between intermediate and other habitats, whether rarefied by area or individuals. Intermediate habitats were the most diverse and sand the least (Fig. 3b,c,e,f). These patterns were consistent whether analyzed based on composite‐area or composite‐individuals replicates (Fig. 3g,h).

### Faunal Composition

**Figure 4:**
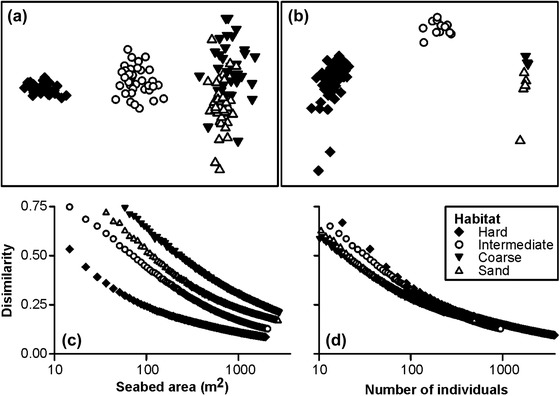
Variation in faunal composition and autosimilarity with habitat type (hard, ≥50% seafloor cover by bedrock, boulder, cobbles; intermediate, ≥10% seafloor cover by bedrock, boulder, cobbles; coarse, >90% seafloor cover by gravelly sand, granules, pebbles, shells; sand, >90% seafloor cover by sand): 2‐dimensional nonmetric multidimensional scaling ordination of Bray‐Curtis dissimilarity of log‐transformed numerical density of combined invertebrates and demersal fish (>=1 cm body length) in (a) composite‐area samples and in (b) composite‐individuals samples and autosimilarity curves plotted by (c) seabed area sampled and (d) number of individuals sampled.

Faunal composition in composite‐area replicates varied significantly with substratum type (ANOSIM, *R* = 0.80, *p* < 0.001). Ordination suggested 3 distinct sample groupings, corresponding with the hard, intermediate, and sedimentary habitats (Fig. 4a), that were ordered by the relative occurrence of hard substratum. Within each of these 3 primary groups, samples were also well ordered by the relative occurrence of coarse and sand substrata (Supporting Information). All pairwise comparisons of faunal composition by substratum type were statistically significant (ANOSIM *R* = 0.36–1.00, *p* < 0.05) (Supporting Information). Indicator species analysis suggested numerous taxa as statistically significant indicators for hard habitats, single taxa for the intermediate and coarse habitats, and 3 taxa for the sand habitats (Table 2 & Supporting Information). Two‐way indicator species analysis (TWINSPAN) almost perfectly divided the samples into the visually determined summary‐habitat classes, on the basis of faunal composition alone. All hard (*n* = 26), intermediate (35), and coarse (33) samples were correctly classified; 4 of the 36 sand samples were misclassified as coarse (Table 2).

**Table 2:** Summary of indicator species analyses conducted on composite‐area samples.^a^

Two‐way indicator species analysis (TWINSPAN; Hill 1979)					Indicator species analysis (indicspecies R package; De Cáceres & Legendre 2009)				
						frequency of occurrence by summary habitat type (%)^c^			
D1 sample group^b^	Taxon	D2 sample group^b^	Taxon	no. of samples in group^c^	taxon	H	I	C	S
1	Bryozoa 01* Porifera 23 Axinellidae spp.	1.1	Axinellidae spp.* *Porella* sp. *Parazoanthus* sp.	26 × H	*Parazoanthus* sp.*	100.0	34.3	3.0	0.0
					Axinellidae spp.*	100.0	77.1	0.0	0.0
					*Porella* sp.*	100.0	74.3	0.0	0.0
					Porifera 20*	100.0	28.6	0.0	0.0
					Salmacina dysteri*	100.0	65.7	0.0	5.6
					*Munida* sp.*	100.0	74.3	3.0	5.6
					Echinus esculentus*	96.2	37.1	0.0	0.0
					*Reteporella* spp.*	100.0	40.0	0.0	5.6
					Stichastrella rosea*	100.0	60.0	27.3	19.4
					*Antedon* spp.*	80.8	28.6	3.0	2.8
		1.2	Anthozoa 34 Anthozoa 39 *Lepidorhombus whiffiagonis*	35 × I	Anthozoa 39*	7.7	42.9	12.1	2.8
2	Perciforme spp. 10 Gadidae spp. Paguridae 02	2.1	Bolocera spp.* Anthozoa 16	33 × C 4 × S	Paguridae 01*	3.8	0.0	21.2	8.3
		2.2	Paguridae 02* Cerianthid 01* Anthozoa 03*	32 × S	Perciforme spp. 10*	0.0	5.7	6.1	33.3
					*Liocarcinus* spp.*	0.0	5.7	3.0	25.0
					Hippoglossoides platessoides*	3.8	2.9	0.0	19.4

a Asterisk: Statistically identified indicator taxa and preferentially occurring taxa (limited to 10 examples from hard summary habitat).

b First (D1) and second (D2) hierarchical divisions of TWINSPAN ordination‐based divisive sample classification.

c Summary habitat types: hard, ≥50% seafloor cover by bedrock, boulder, cobbles; intermediate, ≥10% seafloor cover by bedrock, boulder, cobbles; coarse, >90% seafloor cover by gravelly sand, granules, pebbles, shells; sand, >90% seafloor cover by sand.

Faunal composition in composite‐individuals replicates also showed very clear groupings, corresponding with the hard, intermediate, and sedimentary habitats, and separation of the coarse and sand habitats (Fig. 4b). All pairwise comparisons of faunal composition between habitats were statistically significant with strong differentiation in most comparisons (ANOSIM *R* = 1.0, *p* ≤ 0.002), except between coarse and sand, which were nonetheless statistically significant (ANOSIM *R* = 0.53, *p* = 0.036). Autosimilarity curves for the 4 summary habitats varied considerably when assessed in terms of seabed area sampled (Fig. 4c). That variability was substantially reduced when assessed in terms of the number of individuals sampled (Fig. 4d), reflecting the major difference in faunal density between habitats (e.g., Fig. 2). To achieve a target assemblage description level of 0.75 self‐similarity, composite‐area samples would vary from 90 to 1840 m^2^ between habitats or from 140 to 220 specimens per composite‐individuals sample.

## Discussion

The area surveyed was characterized by the presence of sand and coarser‐grained sedimentary environments, together with outcropping bedrock, boulder, and cobbles substrata. We believe the variety and complexity of the physical environment of the Greater Haig Fras marine conservation zone represents a good test area for the conservation assessment of other large MPAs. From an ecological perspective, the presence of hard substrata exerted a strong positive control on faunal density, biomass, and total species richness; mosaic habitats substantially enhanced faunal diversity; and all primary habitats and mosaics supported distinct faunal assemblages. Photographic assessment provided a uniform field‐ and data‐analysis method across rocky and sedimentary habitats that enabled us to make a direct assessment of multiple biotopes and their occurrence in mosaic form. This ability to resolve ecologically significant information, at broad scale, across multiple and mixed habitats, suggests that the AUV‐based photographic survey was an effective and efficient practical conservation tool in the present case and indicated its potential value in other similarly complex marine habitats.

### Mosaic Habitats

Intermediate habitats, or mosaics of hard substratum within a sedimentary matrix, represented 1‐quarter of the seafloor area observed. Their ecological characteristics were largely predictable as an admixture of their component habitats and consistent with a simple ecotone concept (Odum & Barrett 2005). Faunal density in intermediate habitats was significantly different from, and transitional to, both hard and sedimentary habitats. Regardless of whether rarefied by individuals or seabed area, heterogeneity diversity measures were significantly elevated in intermediate habitats over both hard and sedimentary habitats. This suggests that the addition of the 2 assemblages (i.e., hard and sedimentary) acted to reduce the dominance component of diversity in the combined assemblage.

When taxon richness was assessed as species density (Whittaker et al. 2001), intermediate habitats were significantly different from, and transitional to, both hard and sedimentary habitats (hard > intermediate > coarse ∼ sand). However, when assessed as number of species per individual, the habitats were not statistically distinct and were ordered differently (intermediate > coarse > hard > sand). Species density and total faunal density exhibited the same pattern and might both be controlled by resource availability. In contrast, heterogeneity diversity appeared to exhibit a different pattern related to seafloor‐habitat complexity: uniform sediment (sand) < mixed sediment (coarse) < topographically complex cobbles, boulder, or bedrock (hard) < mosaicked hard substratum islands in a sedimentary matrix (intermediate). Environmental heterogeneity is thought to be a key driver of species richness (Yang et al. 2015), as was evident in our study, although the effect was more pronounced in the case of heterogeneity diversity.

Mosaic habitats are thought to play a key role in the connectivity of marine ecosystems, in terms of both secondary productivity and the maintenance of biological diversity (Olds et al. 2016). They can represent corridors, or stepping‐stones, facilitating the movement of organisms and thereby facilitating processes between dispersed primary habitats. In the case of Haig Fras, the SAC protects what is thought to be the only substantial area of offshore rocky reef habitat in the Celtic Sea. The substantial presence of mosaic habitats in our survey area, and more widely in the Celtic Sea (Thompson et al. 2017), indicates both the potential connectivity of dispersed rocky reefs in the region and the need to protect some of that mosaic habitat in the background environment. These observations provide strong support for the calls to both record (classify) and quantify these mosaic habitats (e.g., Galparsoro et al. 2012; Dauvin 2015). There is also an obvious need to define the physical scale at which the occurrence of mosaics is practically assessed and at which conservation policies might be applied. The quality of the intervening matrix environment may determine the effectiveness of connectivity (Baum et al. 2004) and has been a matter of concern in terrestrial conservation schemes (Donald & Evans 2006).

### Practical Conservation

The United Kingdom has implemented over 200 MPAs; over 27 million km^2^ of MPA have been designated globally (UNEP‐WCMC and IUCN 2019). The routine monitoring of such a large network implies substantial financial costs. We consider that AUV‐based assessment offers a cost‐effective solution (Wynn et al. 2014). Our survey can be approximated as a 20‐km track accomplished at 1.38 m/s (2.7 knots) (i.e., approximately 4‐h duration). Fitting an identical camera and image storage system to a towed platform, or remotely operated vehicle, and operating at 0.26 m/s (0.5 knots), the survey would require at least 21 h of ship time. In the case of a towed platform, sea state (swell waves) can be expected to render about 25% of images unusable. Therefore, the effective survey speed is 0.20 m/s, and the full survey would require at least 28 h of ship time. Consequently, in the case of our survey, the AUV‐based approach offers a potential 86% saving on ship‐time cost or carbon footprint compared with an equivalent towed‐camera survey, and perhaps a 96% saving if the ship carries out other useful work for 3 h while the AUV is submerged.

In terms of cost effectiveness and conservation effectiveness, survey design may be a key factor, raising 2 fundamental questions: what sampling unit is required to obtain suitably accurate and precise data (Galparsoro et al. 2012) and how should the survey be conducted (Foster et al. 2014)? Our study demonstrates that AUV photography can provide enhanced information on the nature of the substratum and its associated fauna. The distribution of the identified habitat types closely matched the sidescan sonar mapping, suggesting consistency and accuracy in the visual assessment method. That we were able to detect statistically significant differences in the key ecological parameters (standing stock, species richness and diversity, faunal composition, and indicator taxa) suggests the technique can produce suitably robust data. Visual monitoring also provided direct evidence of human impacts in the form of lost or discarded fishing gear and plastic debris at the seabed (Supporting Information).

Although our survey was undertaken in a fixed‐grid form suited to the complete bathymetric and sidescan sonar mapping of the area, our subsequent treatment of the photographic data changed the character of the biological survey. By partitioning the seafloor into substratum types and then randomly forming sampling units within those types, we converted the nonrandom grid survey to a form of a posteriori stratified random sampling scheme. We were able to visually identify seafloor habitat type at a much smaller physical scale (1 m^2^) than we think is necessary to appropriately sample the associated fauna (≥150 m^2^). This point may be particularly important in the development of cost‐effective monitoring for complex marine habitats.

There are many potential options for AUV survey design (Foster et al. 2014); however, their implementation may require prior knowledge of environmental stratification and (or) the appropriate sampling unit. Consequently, the combined a posteriori stratification and composite sampling that we have adopted here may have broad, cost‐effective, general application in many marine systems, perhaps particularly in spatially complex environments (Huvenne et al. 2011; Thornton et al. 2016). Our approach is potentially applicable to any image‐data set that can be partitioned into ecologically relevant subsets based on some known or identifiable environmental variable or variables. For example, Morris et al. (2016) segregated their data by topographic height to contrast the ecology of a small abyssal hill with that of the surrounding plain (northeast Atlantic); Simon‐Lledó et al. (2019c) assessed ecological variation over a manganese nodule occurrence gradient in the Clarion‐Clipperton Zone (northeast Pacific), partitioning their data by seafloor nodule coverage with an automated detection technique (Schoening et al. 2017); and Simon‐Lledó et al. (2019b) assessed the long‐term impact of simulated deep‐sea mining in the Peru Basin (southeast Pacific) by segregating their data on proximity to 26‐year‐old seabed plough marks.

Our results suggest that parameters of conservation value exhibit various responses to the choice of sampling unit, primarily linked to the number of specimens encompassed. Numerical density was essentially insensitive to unit size (Fig. 1c), contrary to biomass density that was highly sensitive to unit size (Fig. 1d). Bett (2019) examined how estimated biomass may vary with sampling‐unit size given a power‐law distribution of individual body sizes. We found that estimated species richness was linked to sampling unit size (Fig. 3a,d), as have many previous authors (e.g., Sanders 1968; Colwell et al. 2012), and that similarly faunal composition was substantially influenced by unit size (Fig. 4). In the case of biomass and species richness, unit size had a direct impact on the value (accuracy) of the measured parameter. In the case of faunal composition, unit size affected the variability (precision) of resulting assessments (i.e., the ability to define, discriminate, or monitor the status of a given assemblage or biotope). Simon‐Lledó et al. (2019a) reached similar conclusions in their assessment of the effect of sampling‐unit size on the description of deep‐sea megabenthos assemblages based on AUV photography.

Anderson and Santana‐Garcon (2015) tackled the issue of variability in faunal composition in a manner similar to ours. They pooled subsamples and asked how many original smaller‐scale sampling units were needed to provide a reasonable measure of community structure for comparative analysis. Defining what is reasonable is likely to require case‐by‐case consideration of specific survey objectives. Forcino et al. (2015) considered the appropriate minimum number of specimens per sampling unit across a broad range of terrestrial and aquatic community types. They suggest a minimum number of 58 individuals per unit as adequate for multivariate analyses. However, they note that number is likely to be higher if assemblage evenness and taxon richness are high and ecological contrasts (in space or time) are low.

We based our assessment of the appropriate number of individuals per sampling unit on a target within‐habitat dissimilarity between replicates of 0.25, yielding a range of approximately 150–250 individuals per composite sample across habitats. We aimed to standardize sampling effort between habitat‐specific samples by equalizing dissimilarity between samples within the habitats of interest, rather than simply standardizing by seabed area examined. At a more complex level, an optimized data‐analysis strategy could potentially entail habitat‐based rules, in our case: sand ≥150 and hard ≥250 individuals per composite sample.

Whether based on the autosimilarity curve approach we have adopted or the assessment of multivariate dissimilarity‐based standard error developed by Anderson and Santana‐Garcon (2015), we suggest users consider the potential value of defining their sampling units in terms of number of individuals rather than automatically adopting an area‐defined unit. We suspect this approach may have broad application in marine conservation studies, particularly those based on photographic assessments, and should be simple to implement for mass photography from both ROVs and AUVs. We recognize this may represent a substantial departure from standard practice; nevertheless, we suggest users consider the potential benefits to their own conservation‐status assessment and monitoring objectives. To produce reliable comparative assessments of marine benthic diversity, the number of individuals examined needs to be controlled (Sanders 1968), and this requirement could be valuably expanded to assessments of biomass density and faunal composition.

Marine environmental monitoring and conservation capability is increasing rapidly with the availability of new technology (e.g., Jones et al. 2019). Methods for the automated classification of seafloor images are being developed in the quantification of phytodetritus cover (Morris et al. 2016), the characterization of manganese nodule fields (Schoening et al. 2017), and the identification and coverage estimation of kelp forests (Marzinelli et al. 2015), corals, and macroalgae (Monk et al. 2018). However, the routine widespread use of automated detection and recognition of individual seafloor species occurrences is not yet possible, although progress seems certain in the coming years. We consider that AUVs are a mature technology (several commercial systems are available for photographic and acoustic mapping work) that offer a practical step change in marine conservation capability. The use of mass photography to achieve such aims will, however, require some change in common practices. Given the goals of cost savings per survey and use of a common method across biotopes and habitats, such change may be a key part of achieving a practical means to more effectively monitor the world's growing network of MPAs.

## Supporting information

Seabed type classification (Appendix S1), litter and biological features (Appendix S2), length–weight relationships (Appendix S3), composite‐sample formation (Appendix S4), testing of randomization process (Appendix S5), multivariate analyses of composite‐area samples (Appendix S6), and indicator species (Appendix S7) are available online. The authors are solely responsible for the content and functionality of these materials. Queries (other than absence of the material) should be directed to the corresponding author.
