# Supplementary material for: Monitoring mosaic biotopes in a marine conservation zone by autonomous underwater vehicle
Source: Conserv Biol. 2019 Apr 29;33(5):1174–86. doi: 10.1111/cobi.13312 (PMC6850053; doi:10.1111/cobi.13312)
Supplement: Supplementary file 1 — Seabed type classification (Appendix S1), litter and biological features (Appendix S2), length–weight relationships (Appendix S3), composite‐sample formation (Appendix S4), testing of randomization process (Appendix S5), multivariate analyses of composite‐area samples (Appendix S6), and indicator species (Appendix S7) are available online. The authors are solely responsible for the content and functionality of these materials. Queries (other than absence of the material) should be directed to the corresponding author. [file COBI-33-1174-s001.pdf]

SUPPORTING INFORMATION

Conservation Biology

"Monitoring mosaic biotopes in a marine conservation zone
by autonomous underwater vehicle"

Appendix S1. Example images illustrating visual classification of seabed substratum type.

Appendix S2. Example images of litter, other human debris, and biological features of interest.

Appendix S3. Length-weight relationships used to establish individual biomass.

Appendix S4. Simplified 'cartoon' graphic representation of composite-sample formation.

Appendix S5. Assessment and testing of randomization of composite-sample formation.

Appendix S6. nMDS ordination and ANOSIM of composite-area samples by substratum type.

Appendix S7. Full listing of indicator species analysis.

Appendix S1 Seabed-type classification

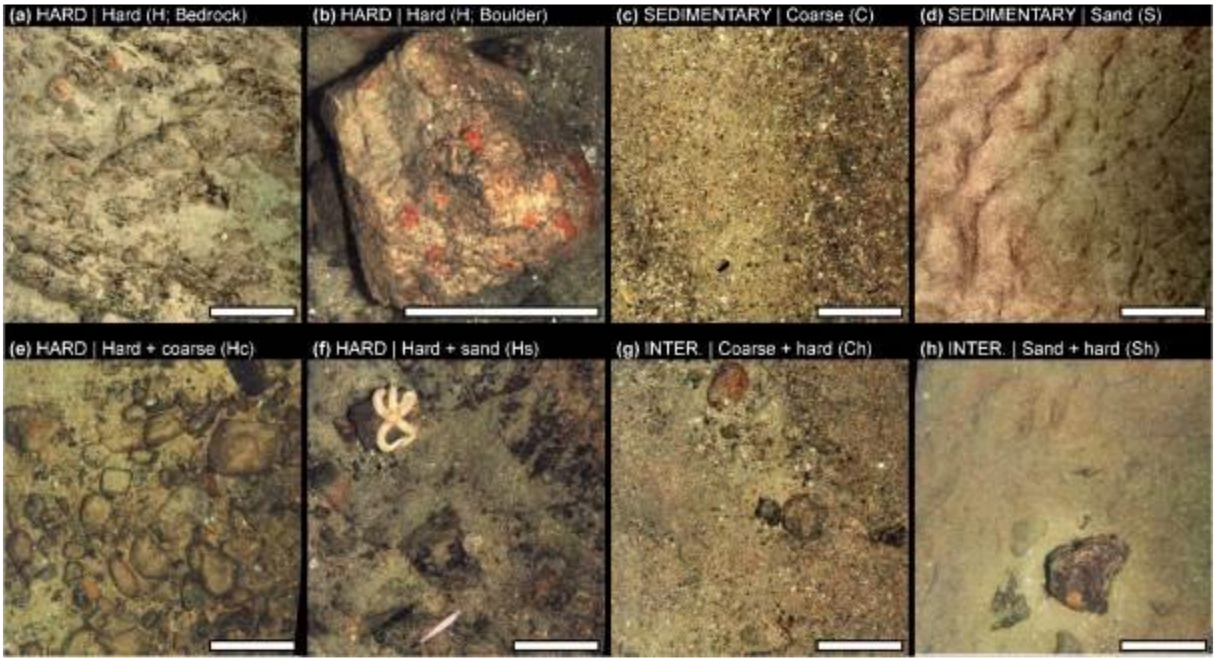

Appendix S1.1. Examples of substratum types. The top pane shows the three main substratum types visually identified: hard substrata with **(a)** bedrock and **(b)** boulder, **(c)** coarse sediments (gravelly sand, granules, pebbles, shells), and **(d)** sand. The bottom pane shows mixed, or mosaic, substratum categories with hard as primary substratum type: **(e)** hard + coarse, **(f)** hard + sand, and hard as secondary substratum type: **(g)** coarse + hard, **(h)** sand + hard. The categories with hard as the only or primary substratum type are referred to as "hard habitats" (a,b,e,f), those with hard as secondary are referred to as "intermediate habitats" (c,g), and those where hard is absent are referred to as "sedimentary habitats" (d,h). Scale bar represents 30 cm.

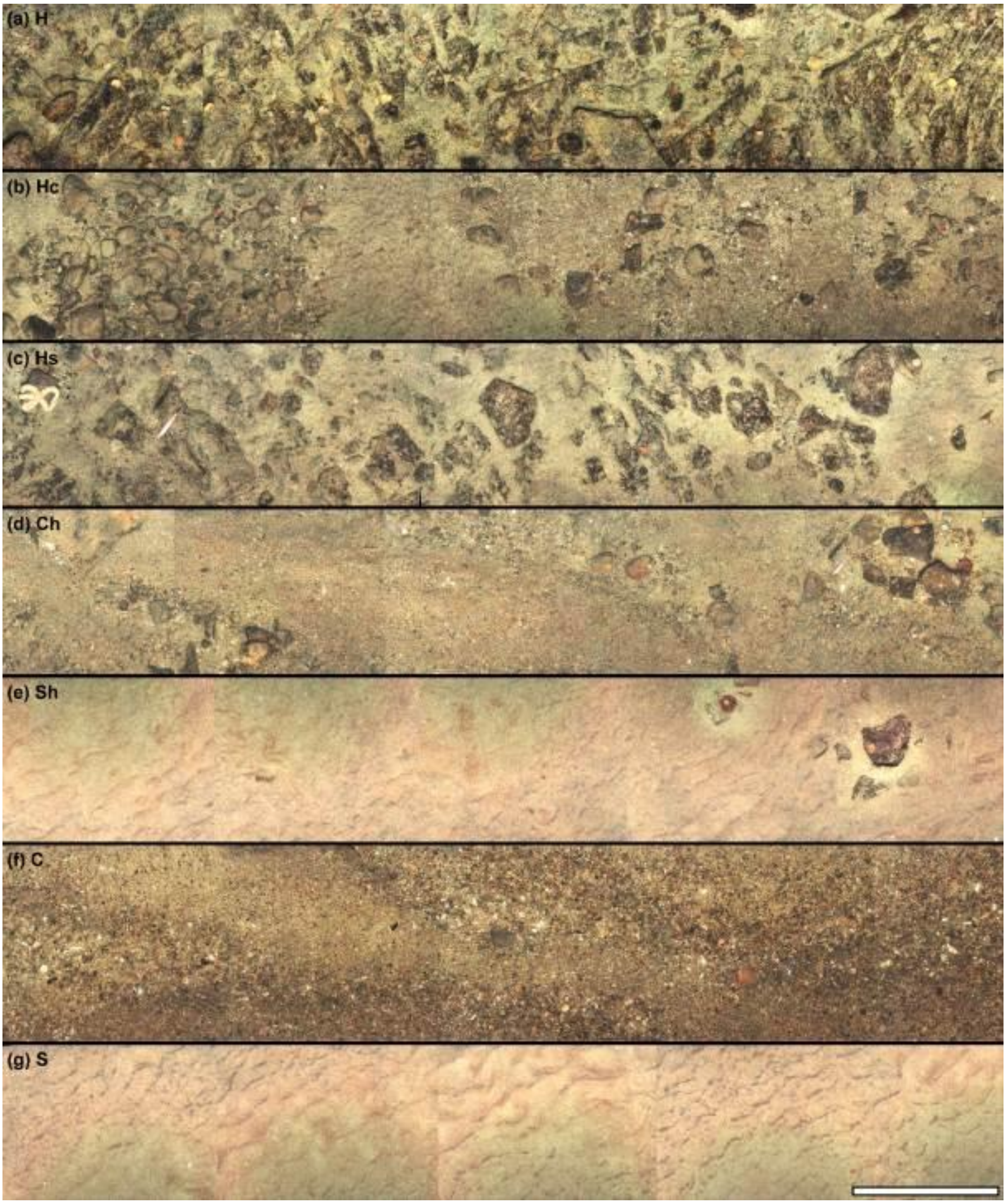

Appendix S1.2. Examples of tile-scale substratum type classification: **(a)** hard, **(b)** hard + coarse, **(c)** hard + sand, **(d)** coarse + hard, **(e)** sand + hard, **(f)** coarse, and **(g)** sand. The categories with hard as the sole or primary substratum type are referred to as hard habitats (a-c), those where it was secondary as intermediate habitats (d,e), and those where it was absent as sedimentary habitats (f,g). Scale bar represents 50 cm.

Appendix S2 Litter and biological features

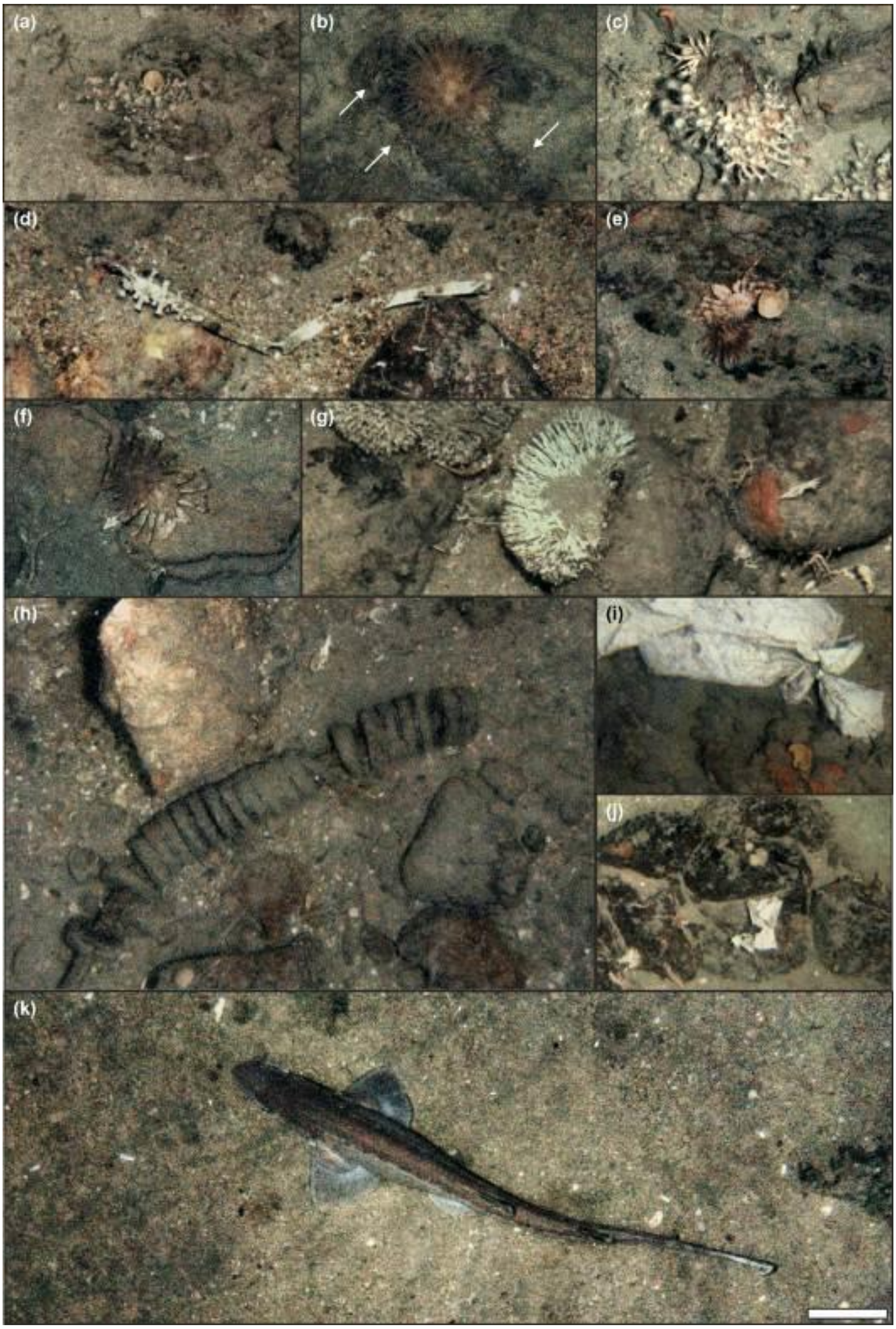

Appendix S2. Examples of close spatial association between distinct taxa **(a-g)**, anthropogenic debris **(h-j)**, and **(k)** rarities [*Galeus* sp.]. **(a)** Axinellidae spp. and *Parazoanthus* sp.; **(b)** *Bolocera* sp. and natant decapods; **(c)** *Parazoanthus* sp. and *Porella* sp.; (d) *Parazoanthus* sp. growing on anthropogenic item; **(e)** *Bolocera* sp., *Lithodes maja*, and Axinellidae spp.; **(f)** *Bolocera* sp. and *Lithodes maja*; **(g)** *Salmacina dysteri* and *Munida* sp.; **(h)** trawl net section; **(i-j)** plastic bag / debris.

Appendix S3 Length-weight relationships

Appendix S3. Specimen body-size measurements were converted from image pixel to seabed units (mm) by trigonometry (Morris et al. 2014). Individual wet weight (g wwt) was estimated from length-weight relationships in the form $W = a\,L^b$, W, gram wet weight (g wwt), L, length (mm), and a and b are coefficients gathered from the literature for each morphotype (or near approximation by taxon and body form). Where a specimen was obscured such that a measurement could not be made, it was assigned the mean individual body weight of the corresponding taxon / morphotype. The body weight of 55 of the 74 morphotypes was estimated, corresponding to 34% of the specimens observed. The unquantified taxa were predominantly colonial or encrusting forms. This table lists all taxa / morphotypes encountered in the Greater Haig Fras marine conservation zone survey, the number of specimens (*n*), the body dimension measured (Dim.), the Range of measurements made, the literature source (Ref.), the reference taxon (Ref. taxon), the coefficients *a* and *b* (Coeff.), and the range of measurements in the literature source (RRange).

Taxon / Morphotype			*n* (ind.)	Dim.	Range (mm)		Ref.	Ref. taxon	Coeff. *a*	Coeff. *b*	RRange (mm)	
Annelida	Polychaeta	*Salmacina dysteri*	547	D	-	-	-	-	-	-	-	-
Arthropoda	Malacostraca	Inachidae 01 (?*Inachus* spp.)	39	Wc	14	34	[1]	*I. dorsettensis*	$1.73 \cdot 10^{-03}$	2.541	5	25
	Malacostraca	Inachidae 02 (?*Macropodia* spp.)*	3	Wc	-	-	-	-	-	-	-	-
	Malacostraca	*Liocarcinus* spp.	9	Wc	18	53	[1]	*L. depurator*	$1.59 \cdot 10^{-04}$	3.144	3	52
	Malacostraca	*Lithodes maja*	14	Wc	27	76	[2]	*L.murrayi*	$5.95 \cdot 10^{-04}$	3.009	4	11
	Malacostraca	Munida spp. (include *M. rugosa*)	480	Lc	9	62	[1]	*M. rugosa*	$8.49 \cdot 10^{-04}$	3.073	6	34
	Malacostraca	Paguridae 01 (*Pagurus* ?*bernhardus*)	14	Wc	29	64	[3]	*P. bouvieri*	$4.60 \cdot 10^{-01}$	1.055	10	27
	Malacostraca	Paguridae 02 (*Pagurus* ?*prideaux*)	625	Wc	16	70	[3]	*P. bouvieri*	$4.60 \cdot 10^{-01}$	1.055	10	27
	Malacostraca	Porcellanidae spp.	2	Wc	35	36	[1]	*L. depurator*	$1.59 \cdot 10^{-04}$	3.144	3	52
Bryozoa	Bryozoa	Bryozoa 01	777	D	-	-	-	-	-	-	-	-
	Gymnolaemata	*Pentapora foliacea*	11	D	-	-	-	-	-	-	-	-
	Gymnolaemata	Porella spp. (*P. ?compressa*)	1504	L	-	-	-	-	-	-	-	-
	Gymnolaemata	*Reteporella* spp.	130	D	-	-	-	-	-	-	-	-
Chordata	Actinopterii	*Callionymus* spp. (include *C. lyra*, *C. maticulatus*)	22	L	50	228	[1]	*C. lyra*	$1.02 \cdot 10^{-05}$	2.927	26	228
	Actinopterii	Fish 10	19	L	36	86	[1]	*D. bimaculata*	$2.59 \cdot 10^{-05}$	2.737	20	36
	Actinopterii	Gadidae spp.	514	L	124	578	[1]	*G. morhua*	$1.21 \cdot 10^{-07}$	3.819	50	550
	Actinopterii	Gadiforme 09 (?*Merluccius merluccius*)	2	L	340	340	[4]	*M. merluccius*	$3.25 \cdot 10^{-06}$	3.099	160	660
	Actinopterii	*Gadus morhua*	1	L	124	578	[1]	*G. morhua*	$1.21 \cdot 10^{-07}$	3.819	50	550
	Actinopterii	*Gaidropsarus vulgaris*	2	L	124	294	[1]	*G. vulgaris*	$3.40 \cdot 10^{-05}$	2.547	26	91
	Actinopterii	*Hippoglossoides platessoides*	13	L	102	274	[1]	*H. platessoides*	$7.41 \cdot 10^{-06}$	2.978	12	250
	Actinopterii	*Lepidorhombus whiffiagonis*	37	L	139	414	[1]	*L. whiffiagonis*	$2.40 \cdot 10^{-05}$	2.746	25	315
	Actinopterii	*Microchirus variegatus*	13	L	126	225	[1]	*M. variegatus*	$5.75 \cdot 10^{-06}$	3.141	22	155
	Elasmobranchi	*Galeus* sp. (*G. ?melastomus*)	1	L	727	727	[5]	*G. melastomus*	$1.73 \cdot 10^{-06}$	3.020	95	600
	Elasmobranchi	*Leucoraja naevus*	5	Lw	288	489	[4]	*R. naevus*	$2.68 \cdot 10^{-05}$	2.959	80	380
	Elasmobranchi	Rajidae sp. (?*Dipturus* sp.)	2	Lw	480	483	[4]	*R. naevus*	$2.68 \cdot 10^{-05}$	2.959	80	380
	Elasmobranchi	*Scyliorhinus canicula**	2	L	-	-	-	-	-	-	-	-
Cnidaria	Anthozoa	Anthozoa 01	35	CD	10	43	[6]	Cnidaria	$2.60 \cdot 10^{-03}$	2.360	5	80
	Anthozoa	Anthozoa 03	163	CD	10	68	[6]	Cnidaria	$2.60 \cdot 10^{-03}$	2.360	5	80
	Anthozoa	Anthozoa 05 (?*Sagartia* spp.)	14	CD	19	236	[6]	Cnidaria	$2.60 \cdot 10^{-03}$	2.360	5	80
	Anthozoa	Anthozoa 06	22	CD	17	39	[6]	Cnidaria	$2.60 \cdot 10^{-03}$	2.360	5	80
	Anthozoa	Anthozoa 07	1	CD	54	54	[6]	Cnidaria	$2.60 \cdot 10^{-03}$	2.360	5	80
	Anthozoa	Anthozoa 08	35	CD	8	38	[6]	Cnidaria	$2.60 \cdot 10^{-03}$	2.360	5	80
	Anthozoa	Anthozoa 11	35	CD	15	119	[6]	Cnidaria	$2.60 \cdot 10^{-03}$	2.360	5	80
	Anthozoa	Anthozoa 16	32	CD	12	43	[6]	Cnidaria	$2.60 \cdot 10^{-03}$	2.360	5	80
	Anthozoa	Anthozoa 19	2	CD	36	57	[6]	Cnidaria	$2.60 \cdot 10^{-03}$	2.360	5	80
	Anthozoa	Anthozoa 21	2	CD	17	17	[6]	Cnidaria	$2.60 \cdot 10^{-03}$	2.360	5	80
	Anthozoa	Anthozoa 24	6	CD	28	49	[6]	Cnidaria	$2.60 \cdot 10^{-03}$	2.360	5	80
	Anthozoa	Anthozoa 34	52	CD	12	54	[6]	Cnidaria	$2.60 \cdot 10^{-03}$	2.360	5	80

**Taxon / Morphotype**			***n* (ind.)**	**Dim.**	**Range (mm)**		**Ref.**	**Ref. taxon**	**Coeff. *a***	**Coeff. *b***	**RRange (mm)**	
	Anthozoa	Anthozoa 39	26	CD	19	53	[6]	Cnidaria	$2.60\cdot10^{-03}$	2.360	5	80
	Anthozoa	*Bolocera* spp. (include *B. tuediae*)	251	CD	13	70	[6]	Cnidaria	$2.60\cdot10^{-03}$	2.360	5	80
	Anthozoa	*Caryophyllia smithii**	379	CD	-	-	-	-	-	-	-	-
	Anthozoa	Cerianthid 01 (?*Arachnanthus* sp.)	181	CD	14	78	[6]	Cnidaria	$2.60\cdot10^{-03}$	2.360	5	80
	Anthozoa	Cerianthid 03 (?*Pachycerianthus* spp.)	16	CD	27	64	[6]	Cnidaria	$2.60\cdot10^{-03}$	2.360	5	80
	Anthozoa	Hormathiid 01 (?*Actinauge* spp.)	3	CD	23	44	[6]	Cnidaria	$2.60\cdot10^{-03}$	2.360	5	80
	Anthozoa	*Parazoanthus* (*P. ?anguicomus*)	1629	D	-	-	-	-	-	-	-	-
	Anthozoa	*Urticina* spp. (include *U. felina*)	27	CD	21	91	[6]	Cnidaria	$2.60\cdot10^{-03}$	2.360	5	80
	Hydrozoa	Hydroid 01 (?*Abietinaria* spp.)	16	L	-	-	-	-	-	-	-	-
Echinodermata	Asteroidea	*Asterias rubens*	16	L/D	46	122	[1]	*A. rubens*	$3.59\cdot10^{-04}$	2.509	1	280
	Asteroidea	Asteroid 01	17	L/D	15	38	[1]	*P. pulvillus*	$3.15\cdot10^{-04}$	2.706	8	101
	Asteroidea	Asteroid 02 (?*Henricia oculata*)	16	L/D	8	26	[1]	*H. sanguinolata*	$3.99\cdot10^{-04}$	2.350	7	127
	Asteroidea	*Astropecten irregularis*	5	L/D	68	99	[1]	*A. irregularis*	$1.71\cdot10^{-04}$	2.746	3	125
	Asteroidea	*Crossaster papposus*	1	L/D	111	111	[1]	*C. papposus*	$2.85\cdot10^{-05}$	3.144	8	167
	Asteroidea	*Luidia ciliaris*	25	D	58	260	[1]	*L. sarsii*	$1.10\cdot10^{-04}$	3.940	4	99
	Asteroidea	*Luidia sarsii*	3	D	53	77	[1]	*L. sarsii*	$1.10\cdot10^{-04}$	3.940	4	99
	Asteroidea	*Marthasterias glacialis*	2	L/D	119	119	[1]	*A. rubens*	$3.59\cdot10^{-04}$	2.509	1	280
	Asteroidea	*Porania pulvillus*	1124	L/D	9	73	[1]	*P. pulvillus*	$3.15\cdot10^{-04}$	2.706	8	101
	Asteroidea	*Stichastrella rosea*	139	L/D	12	114	[1]	*S. rosea*	$3.36\cdot10^{-04}$	2.437	14	112
	Crinoidea	*Antedon* spp. (includes *A. bifida*)	58	L/D	-	-	-	-	-	-	-	-
	Echinoidea	Echinoid 01	15	D	22	102	[1]	*E. acutus*	$5.68\cdot10^{-04}$	2.846	5	79
	Echinoidea	Echinoid 05	4	D	27	33	[1]	*E. acutus*	$5.68\cdot10^{-04}$	2.846	5	79
	Echinoidea	*Echinus esculentus*	128	D	32	124	[1]	*E. esculentus*	$3.47\cdot10^{-04}$	3.012	10	100
	Ophiuroidea	Ophiuroid 01 (include ?*Ophiura* spp.)	207	D	160	207	[1]	*O. sarsi*	$4.52\cdot10^{-03}$	2.216	4	31
	Ophiuroidea	Ophiuroid 02 (include ?*Ophiothrix fragilis*, ?*Ophiocomina nigra*)	13	D	54	102	[1]	*O. nigra*	$5.73\cdot10^{-03}$	2.220	5	11
Mollusca	Cephalopoda	*Eledone cirrhosa*	2	Lm	111	111	[1]	*E. cirrhosa*	$1.95\cdot10^{-03}$	2.672	37	96
	Cephalopoda	*Eledone* 02	3	Lm	57	140	[1]	*E. cirrhosa*	$1.95\cdot10^{-03}$	2.672	37	96
Porifera	Desmospongiae	Axinellidae spp. (include *Phakellia ventilabrum*, *Axinella infundibuliformis*)	2457	D	-	-	-	-	-	-	-	-
	Porifera	Porifera 02	403	L	-	-	-	-	-	-	-	-
	Porifera	Porifera 03	23	D	-	-	-	-	-	-	-	-
	Porifera	Porifera 13	26	L	-	-	-	-	-	-	-	-
	Desmospongiae	Porifera 20 (include ?*Suberites carnosus*)	116	D	-	-	-	-	-	-	-	-
	Desmospongiae	Porifera 22 (?*Quasillina* sp.)	2	D	-	-	-	-	-	-	-	-
	Desmospongiae	Porifera 23 (include ?*Polymastia boletiformis*)	362	D	-	-	-	-	-	-	-	-
	Desmospongiae	Porifera 24 (?*Polymastia* sp.)	7	D	-	-	-	-	-	-	-	-
	Desmospongiae	Porifera 25 (?*Myxilla* sp.)	3	D	-	-	-	-	-	-	-	-

Dimension measured (Dim.): Wc, width of carapace; D, 'longest diameter' of an individual or a colony; CD, column diameter; L, total length; Lc, carapace length; Lm, mantle length; L/D, longest arm to opposite edge of disc. * Partial observation(s) biomass not estimated.

[1] Robinson, L.A., Greenstreet, S.P.R., Reiss, H., Callaway, R., Craeymeersch, J., de Boois, J., Degraer, S., Ehrich, S., Fraser, H.M., Goffin, A., Kröncke, I., Lindal Jorgenson, L., Robertson, M.R., Lancaster, J., 2010. Length-weight relationships of 216 North Sea benthic invertebrates and fish. Journal of the Marine Biological Association of the United Kingdom **90**, 95-104.

[2] Arnaud, P.M., Do-Chi, T., 1977. Données biologiques et biométriques sur les lithodes *Lithodes murrayi* (Crustacea: Decapoda: Anomura) des îles Crozet (SW océan Idien). Marine Biology **39**, 147-159.

[3] Richardson, A.J., Lamberts, C., Isaacs, G., Moloney, C.L., Gibbons, M.J., 2000. Length-weight relationships of some important forage crustaceans from South Africa. Naga, The ICLARM Quarterly **23**, 29-33.

[4] Coull, K.A., Jermyn, A.S., Newton, A.W., Henderson, G.I., Hall, W.B., 1989. Length / weight relationships for 88 species of fish encountered in the North East Atlantic. Scottish Fisheries Research Report Number 43. Department of Agriculture and Fisheries for Scotland, Aberdeen. ISBN 0308 8022.

[5] Merella, P., Quetglas, A., Alemany, F., Carbonell, A., 1997. Length-weight relationship of fishes and cephalopods from the Balearic Islands (Western Mediterranean). Naga, The ICLARM Quarterly **20**, 66-68.

[6] Durden, J.M., Bett, B.J., Horton, T., Serpell-Stevens, A., Morris, K.J., Billett, D.S.M., Ruhl, H.A., 2016. Improving the estimation of deep-sea megabenthos biomass: dimension to wet weight conversions for abyssal invertebrates. Marine Ecology Progress Series **552**, 71-79.

Appendix S4 Composite-sample formation

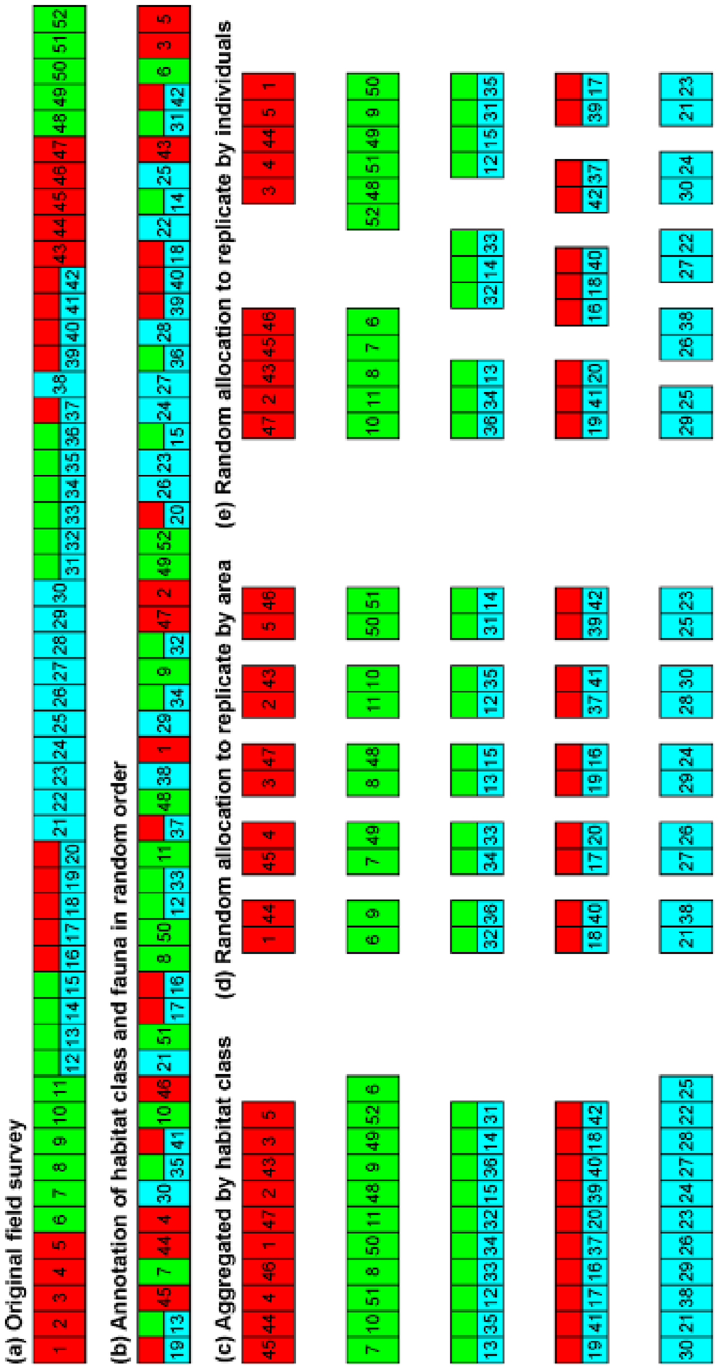

Appendix S4. Simplified 'cartoon' graphic representation of the visual survey method. Note that in our study the field survey comprised 2637 tiles; for clarity, this graphic has only 52 tiles. **(a)** Original non-random spatial survey with substratum type classified by color (e.g., red, sand; green, coarse; cyan, rock; 2-color, mosaic). **(b)** Tiles were examined and annotated for substratum type and faunal composition in random order to avoid systematic temporal annotator bias in habitat classification or faunal identification, and unconscious annotator bias between spatially related images. **(c)** Subsequent aggregation of tiles by substratum type. Then, final random allocation of tile data to replicate composite samples on the basis of: **(d)** approximately fixed seabed area (150 m^2 in our study), or **(e)** approximately fixed number of individuals (150 individuals in our study), to remove the spatial structure (autocorrelation) inherent in the original transect.

Appendix S5 Testing of randomization process

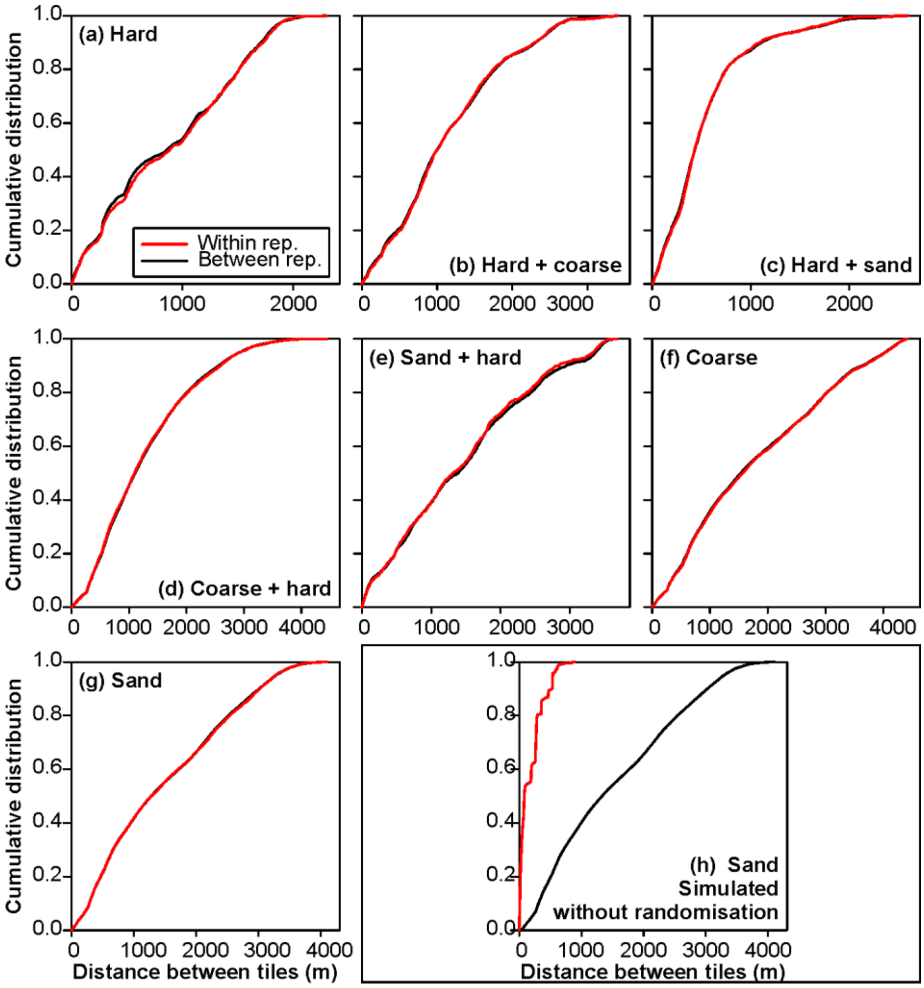

Appendix S5. Testing spatial randomization of photographic tiles. Cumulative distributions of spatial distances between all pairs of tiles within (red) and between (black) replicates. **(a-g)** As analyzed in our study, tiles were randomly selected to compose replicates of approximately 150 m^2 (see Appendix S4). **(h)** A simulated version of the sand dataset produced without randomization, tiles were selected in spatial order to composite replicates of approximately 150 m^2. The close correspondence of within- and between-replicate spatial distances indicates successful randomization (a-g), as contrasted with the obvious disparity in spatial arrangement without randomization (h). The correspondence of within- and between-replicate distance cumulative distributions was assessed by 1-sided (i.e., 'within' not greater than 'between') 2-sample Kolmogorov-Smirnov tests (e.g., Siegel & Castellan 1988), as implemented in R environment with the ks.test function in the R Stats Package (Version 3.5.0) (R Core Team 2017). Results: hard, $D^+ = 0.008$, $p = 0.783$; hard + coarse, 0.014, $p = 0.241$; hard + sand, 0.007, $p = 0.642$; coarse + hard, 0.011, $p = 0.088$; sand + hard, 0.021, $p = 0.202$; coarse, 0.002, $p = 0.91$; sand, 0.004, $p = 0.684$. In all cases analyzed (a-g), cumulative within was not significantly greater than cumulative between, in marked contrast to the simulated (non-randomized) sand dataset where there was a highly significant difference ($D^+ = 0.743$, $p < 0.001$).

Siegel, S, Castellan, NJ, 1988. Nonparametric statistics for the behavioral sciences. New York: McGraw-Hill.
R Core Team, 2017. R: a language and environment for statistical computing. R Foundation for Statistical Computing, Vienna, Austria.

Appendix S6 Multivariate analyses of composite-area samples

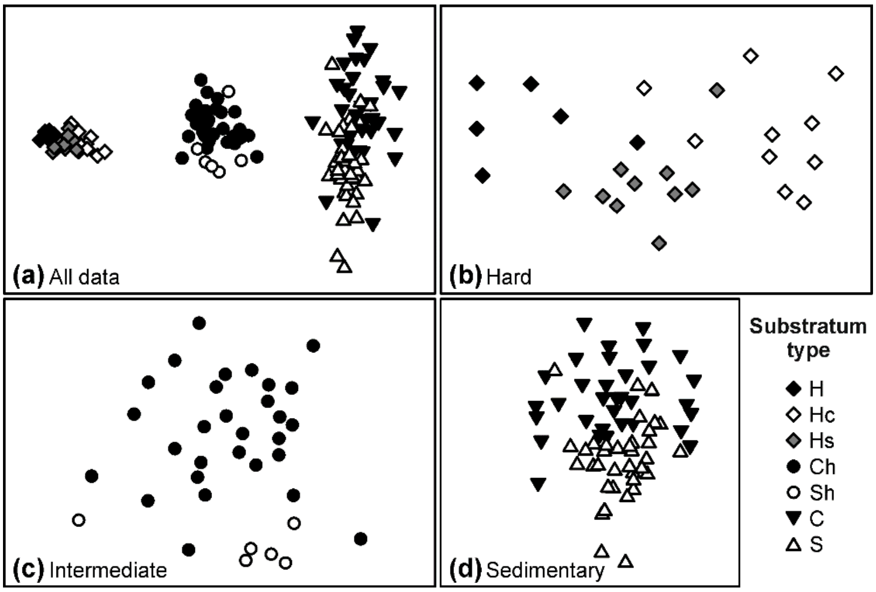

Appendix S6.1. Variation in faunal composition by substratum type in composite-area samples, illustrated by 2-dimensional non-metric multidimensional scaling (nMDS) ordination of Bray-Curtis dissimilarity of log-transformed numerical density. **(a)** All data. **(b)** Hard habitats. **(c)** Intermediate habitats. **(d)** Sedimentary habitats. Primary substratum types are indicated (H)ard, (I)ntermediate, (C)oarse, and (S)and, with secondary substratum types indicated by corresponding lower case letters.

Appendix S6.2. Pairwise comparisons of faunal composition between substratum types in composite-area samples. Lower left half-matrix details ANOSIM *R* values, and upper right half-matrix details average faunal dissimilarity. Primary substratum types are indicated (H)ard, (I)ntermediate, (C)oarse, and (S)and, with secondary substratum types indicated by corresponding lower case letters.

Substratum type	Hard			Intermediate		Sedimentary	
	H	Hc	Hs	Ch	Sh	C	S
H	-	37.9	28.1	83.3	82.0	98.2	98.4
Hc	0.88**	-	29.1	72.1	68.9	95.4	94.8
Hs	0.68**	0.71**	-	78.9	76.5	96.8	96.7
Ch	1.00**	1.00**	1.00**	-	49.4	78.8	77.2
Sh	1.00*	1.00**	1.00**	0.45**	-	79.0	73.5
C	1.00**	1.00**	1.00**	0.94**	0.91**	-	59.2
S	1.00**	1.00**	1.00**	0.96**	0.91**	0.36**	-

* $p < 0.05$, ** $p < 0.001$

Appendix S7 Indicator species

Appendix S7. Indicator species analysis of composite-area samples by summary habitat type: (H)ard, (I)ntermediate, (C)oarse, and (S)and. All taxa with an indicator-value (IndVal[1]) adjusted[2] *p*-value (Adj-*p*) < 0.05 are listed. In each case, specificity (A parameter[1]), fidelity (B parameter[1]), and resultant indicator value are given. Frequency of occurrence (i.e., fidelity) is also listed for each habitat.

Habitat type	Taxon	Specificity	Fidelity	IndVal	Adj-*p*	Frequency (%)			
						H	I	C	S
H	*Parazoanthus* sp.	0.9881	1.0000	0.994	0.0002	100.0	34.3	3.0	0.0
	Axinellidae spp.	0.9671	1.0000	0.983	0.0002	100.0	77.1	0.0	0.0
	Porella sp.	0.9552	1.0000	0.977	0.0002	100.0	74.3	0.0	0.0
	Porifera 20	0.9211	1.0000	0.960	0.0002	100.0	28.6	0.0	0.0
	Salmacina dysteri	0.9181	1.0000	0.958	0.0002	100.0	65.7	0.0	5.6
	Munida sp.	0.9036	1.0000	0.951	0.0002	100.0	74.3	3.0	5.6
	Echinus esculentus	0.9042	0.9615	0.932	0.0002	96.2	37.1	0.0	0.0
	Reteporella spp.	0.8543	1.0000	0.924	0.0002	100.0	40.0	0.0	5.6
	Stichastrella rosea	0.7002	1.0000	0.837	0.0002	100.0	60.0	27.3	19.4
	Antedon spp.	0.8078	0.8077	0.808	0.0002	80.8	28.6	3.0	2.8
	Caryophyllia smithii	1.0000	0.5385	0.734	0.0002	53.8	0.0	0.0	0.0
	Inachidae 01	0.8065	0.5769	0.682	0.0002	57.7	14.3	6.1	0.0
	Porifera 03	0.9673	0.4231	0.640	0.0002	42.3	2.9	0.0	0.0
	Lithodes maja	0.9453	0.4231	0.632	0.0002	42.3	2.9	0.0	0.0
	Porifera 13	0.9056	0.4231	0.619	0.0002	42.3	8.6	0.0	0.0
	Luidia ciliaris	0.6788	0.5000	0.583	0.0002	50.0	11.4	9.1	0.0
	Asterias rubens	0.9474	0.3462	0.573	0.0002	34.6	2.9	0.0	0.0
	Asteroid 07	0.8487	0.3462	0.542	0.0002	34.6	5.7	0.0	2.8
	Cerianthid 03	0.7294	0.3846	0.530	0.0002	38.5	8.6	3.0	2.8
	Asteroid 01	0.7030	0.3846	0.520	0.0004	38.5	8.6	0.0	2.8
	Pentapora foliacea	1.0000	0.2692	0.519	0.0002	26.9	0.0	0.0	0.0
	Hydroid 01	1.0000	0.2308	0.480	0.0002	23.1	0.0	0.0	0.0
	Ophiuroid 02	0.7443	0.2692	0.448	0.0024	26.9	8.6	0.0	2.8
	Echinoid 01	0.6523	0.2692	0.419	0.0062	26.9	11.4	3.0	0.0
	Porifera 24	0.7491	0.1923	0.380	0.0094	19.2	5.7	0.0	0.0
	Porifera 25	1.0000	0.1154	0.340	0.0137	11.5	0.0	0.0	0.0
I	Anthozoa 39	0.7116	0.4286	0.552	0.0002	7.7	42.9	12.1	2.8
C	Paguridae 01	0.6476	0.2121	0.371	0.0322	3.8	0.0	21.2	8.3
S	Fish 10	0.7306	0.3333	0.494	0.0006	0.0	5.7	6.1	33.3
	Liocarcinus spp.	0.7623	0.2500	0.437	0.0033	0.0	5.7	3.0	25.0
	Hippoglossoides platessoides	0.7593	0.1944	0.384	0.0094	3.8	2.9	0.0	19.4

[1] Notation as given by Dufrêne and Legendre (1997), calculated in R environment with the multipatt function in the R indicspecies Package (R Core Team 2017).

[2] Adjustment for multiple testing by method of Benjamini and Hochberg (1995), as implemented in R environment with the p.adjust function in the R Stats Package (Version 3.5.0) (R Core Team 2017).

Benjamini, Y., Hochberg, Y., 1995. Controlling the false discovery rate: a practical and powerful approach to multiple testing. *Journal of the Royal Statistical Society Series B*, **57**, 289-300.

Dufrêne, M., Legendre, P., 1997. Species assemblages and indicator species: the need for a flexible asymmetrical approach. *Ecological Monographs*, **67**, 345-366.

R Core Team, 2017. R: a language and environment for statistical computing. R Foundation for Statistical Computing, Vienna, Austria.
